# A Sir2-Like Protein Participates in Mycobacterial NHEJ

**DOI:** 10.1371/journal.pone.0020045

**Published:** 2011-05-26

**Authors:** Zhongdao Li, Jikai Wen, Yaning Lin, Shihua Wang, Peng Xue, Zhiping Zhang, Ying Zhou, Xiao Wang, Li Sui, Li-Jun Bi, Xian-En Zhang

**Affiliations:** 1 State Key Laboratory of Virology, Wuhan Institute of Virology, Chinese Academy of Sciences, Wuhan, China; 2 Graduate School, Chinese Academy of Sciences, Beijing, China; 3 School of Biosciences, University of Birmingham, Edgbaston, Birmingham, United Kingdom; 4 College of Life Sciences, Fujian Agriculture and Forestry University, Fuzhou, China; 5 National Laboratory of Biomacromolecules and Proteomics Platform, Institute of Biophysics, Chinese Academy of Sciences, Beijing, China; 6 Department of Nuclear Physics, China Institute of Atomic Energy, Beijing, China; New England Biolabs, Inc., United States of America

## Abstract

In eukaryotic cells, repair of DNA double-strand breaks (DSBs) by the nonhomologous end-joining (NHEJ) pathway is critical for genome stability. In contrast to the complex eukaryotic repair system, bacterial NHEJ apparatus consists of only two proteins, Ku and a multifunctional DNA ligase (LigD), whose functional mechanism has not been fully clarified. We show here for the first time that Sir2 is involved in the mycobacterial NHEJ repair pathway. Here, using tandem affinity purification (TAP) screening, we have identified an NAD-dependent deacetylase in mycobacteria which is a homologue of the eukaryotic Sir2 protein and interacts directly with Ku. Results from an *in vitro* glutathione S-transferase (GST) pull-down assay suggest that Sir2 interacts directly with LigD. Plasmid-based end-joining assays revealed that the efficiency of DSB repair in a *sir2* deletion mutant was reduced 2-fold. Moreover, the Δ*sir2* strain was about 10-fold more sensitive to ionizing radiation (IR) in the stationary phase than the wild-type. Our results suggest that Sir2 may function closely together with Ku and LigD in the nonhomologous end-joining pathway in mycobacteria.

## Introduction

DNA double-strand breaks (DSBs) are the most lethal form of DNA damage and pose the greatest threat to genomic DNA integrity [1], [2]. They are caused by a variety of endogenous cellular processes and exogenous factors. All organisms have a range of cellular pathways that repair DSBs. Two major pathways, namely homologous recombination (HR) and nonhomologous end-joining (NHEJ), have evolved to repair DSBs and maintain genetic integrity [3], [4], [5], [6]. The latter is utilized in higher eukaryotes and is a complex pathway, involving many components such as DNA-PKcs, the Ku70/80 heterodimer, Ligase IV, XRCC4, Artemis, and XLF/Cernunos [7], [8]. Proteins of this pathway are critical for maintaining mammalian genomic stability.

Compared with the eukaryotic NHEJ pathway, the prokaryotic NHEJ system has only two known components. Reports have indicated that the two core proteins, Ku and LigD, are sufficient for NHEJ repair *in vitro* [9], [10], and transformation of yeast with Ku and LigD can successfully reconstitute NHEJ *in vivo* [9]. LigD is a multifunctional protein composed of polymerase (PolDom), nuclease (NucDom) and ligase (LigDom) domains [11], and each domain can exert its function independently during the process of DNA end joining. Generally, prokaryotic NHEJ is initiated at double-strand breaks by the recruitment of Ku that binds to each of the DNA ends, which will further recruit LigD through its PolDom [11] to the DNA damage site for the DNA end processing and ligation. The PolDom specifically recognizes the 5′ phosphate group [12] and mediates the synapsis before resection, resynthesis, and ligation of DSBs. The NucDom subsequently resects the nonextendable 3′ termini if required, and the PolDom and LigDom will then re-synthesize and ligate the nicks. The core proteins of Ku and LigD were well studied; however, many details of the prokaryotic NHEJ pathway are unclear [13], [14]. Many of the bacteria that possess NHEJ repair apparatus, for example *Mycobacterium tuberculosis* (Mtu), are major human pathogens. They spend much of their life-cycle in host organisms in the stationary phase, and their DNA repair capacity plays a crucial role in resisting the host response to infection, suggesting that they may rely on NHEJ during prolonged periods of no homologous recombination when homologous templates are lacking [15], [16]. It has been reported that the *Mycobacterium smegmatis* (Msm) Δ*ku* mutant is significantly more sensitive to IR during the stationary phase than the wild-type, providing further evidence of the importance of the NHEJ pathway for survival [17]. Recently, Sinha *et al* [18] found a novel Ku-binding partner of UvrD1 and identified its role in DSB repair in mycobacteria. Mycobacterial UvrD1, a DNA-dependent ATPase I and helicase II, also participates in the nucleotide excision repair (NER) pathway [19] which indicates that some DNA repair proteins might participate in more than one process. It seems likely, therefore, that there may be more additional NHEJ components in mycobacteria yet to be discovered.

Tandem affinity purification (TAP) combined with mass spectrometry has been demonstrated to be an effective and reliable strategy for identifying and purifying protein complexes under native conditions in different organisms [20], [21]. It is a generic two-step affinity purification protocol for isolating TAP-tagged proteins together with their associated proteins. The yeast-based TAP procedure [22] for isolating protein complexes makes use of site-specific recombination to introduce a dual-tagging cassette into specific chromosomal loci. Mycobacteria do not readily recombine exogenous linear DNA fragments into their chromosomes due to their high rate of illegitimate recombination relative to homologous DNA exchange, but expression of the Che9c, gp60 and gp61 proteins from the mycobacterial recombineering system markedly enhances integration [23]. Here we have applied this system to *M. smegmatis* in order to look for additional components in the mycobacterial NHEJ pathway. We identified Sir2, a novel Ku-binding protein and investigated the interactions between Sir2 and Ku using a glutathione S-transferase (GST) pull-down assay, a common approach for studying protein–protein interactions. As anticipated, we discovered that Sir2 also interacts with LigD, and thus postulate that these three components likely form a ternary complex. Results from the analysis of NHEJ efficiency in a *sir2* mutant show for the first time that Sir2 is involved in mycobacterial NHEJ.

## Results

### Generation and characterization of a knock-in strain expressing the TAP-tagged Ku protein

**Figure 1:**
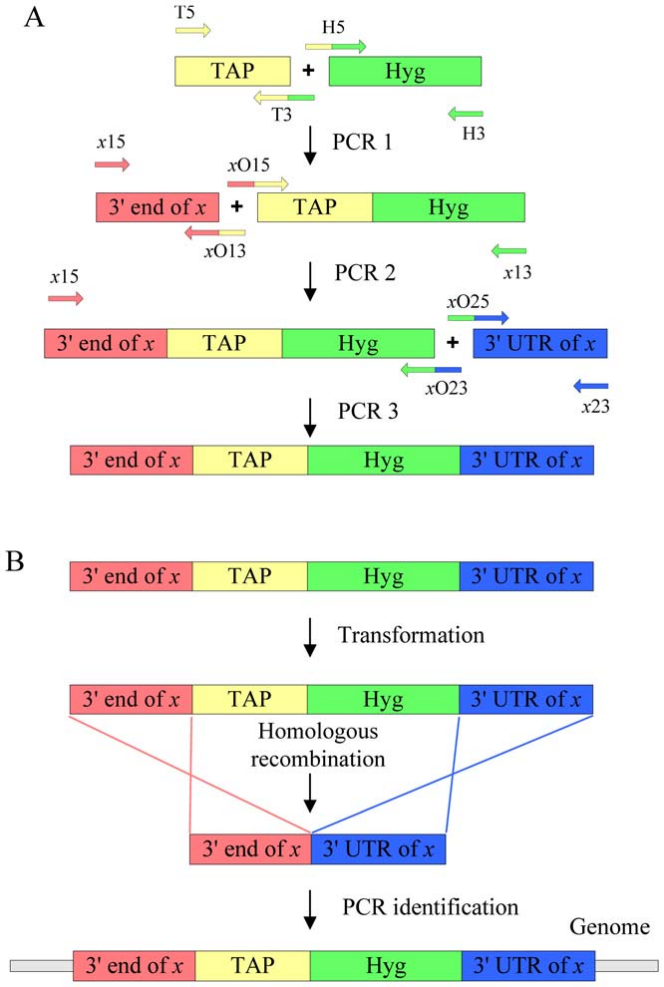
Construction of the TAP-tag knock-in cassette and targeting to a specific gene locus. (A) The TAP-tag knock-in cassette was constructed by three rounds of overlap extension PCR. During the first round of PCR (PCR1), the knock-in core cassette was generated by fusing together the TAP-tag (TAP) gene and the hygromycin (Hyg) gene using four primers T5, T3, H5 and H3 (Table S1). During the following two rounds of PCR, the 3′ end of *x* and 3′ UTR of *x* were inserted adjacent to the N- and C-terminus of the knock-in core fragment during PCR2 and PCR3, respectively, generating the full length TAP-tag knock-in cassette. The primers used in PCR2 and PCR3 are shown in Table S1. *x* indicates the gene to be TAP tagged. (B) The TAP-tag was integrated into the C-terminal coding region of each gene using recombineering methods as described in the materials and methods.

To search for potential components of the mycobacterial NHEJ apparatus, we used TAP combined with mass spectrometry to identify components of protein complexes that interact with Ku. By using overlap extension PCR combined with a mycobacterial homologue recombination system, a TAP-tag knock-in cassette was introduced at the end of the coding region of Ku. The principle is shown in Figure 1. The recombinant strain was screened by PCR (Figure S1) and confirmed by DNA sequencing. Expression of the recombinant Ku protein was verified using a peroxidase anti-peroxidase (PAP) complex antibody that binds the ProtA moiety of the TAP tag (Figure S2). This strain was then used for TAP purification as described below.

### Identification of Sir2 as a new Ku-binding protein by TAP

**Figure 2:**
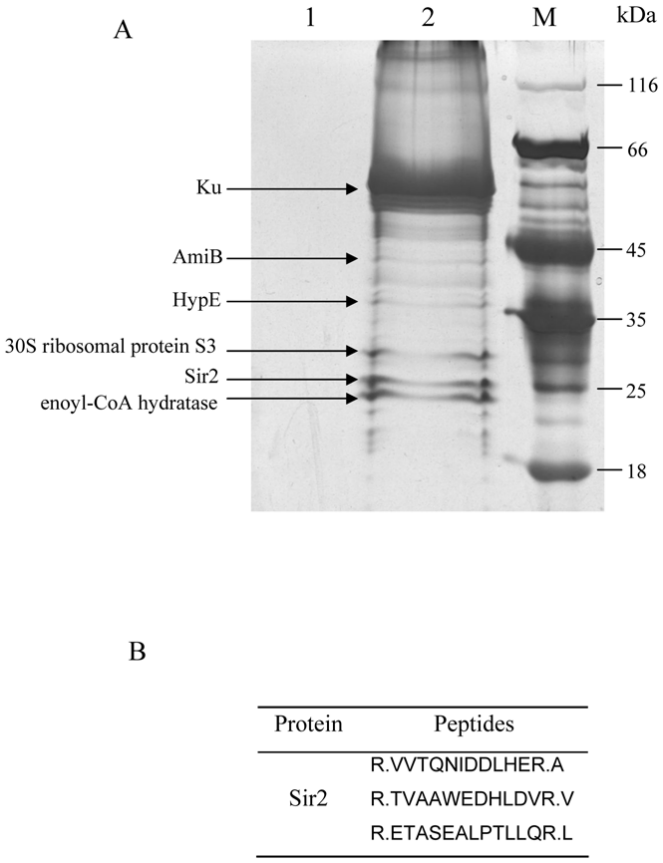
Identification of Sir2 as a Ku-binding protein. (A) Ku (TAP-tagged at the C-terminus) was purified by affinity chromatography with Protein A and calmodulin. Protein complexes were visualized by silver staining after separation by SDS-PAGE. Several specific bands were excised and subjected to mass spectrometry. Lane 1, the TAP tag alone as a control; lane 2, TAP-tagged Ku complex; M, Protein molecular weight marker. (B) The Sir2 peptides were identified by mass spectrometry.

To identify Ku binding partners, we performed TAP in *M. smegmatis* using the constructed Ku-TAP knock-in strain. After separation by SDS-PAGE, Ku binding proteins were visualized by silver staining (Figure 2) and identified by mass spectrometry. Mass spectral data were searched using SEQUEST against NCBI *M. smegmatis* protein database and results were filtered and displayed using the Bioworks 3.2. Of the proteins identified with mass spectrometry, only the protein with more than one peptide identified by mass spectrometry was NAD-dependent deacetylase (MSMEG_5175). Furthermore, an independent replication of the TAP experiment (Figure S3) can only identify this deacetylase protein again. So this protein was selected for subsequent experiments. Protein sequence alignment indicated that NAD-dependent deacetylase was highly homologous with Sir2 family proteins (Figure S4) and it was named MsmSir2. Moreover, the result of a phylogenetic tree analysis revealed that MsmSir2 had more homology with SIRT5 of mammalian Sir2 family than with yeast Sir2 (Figure S4). Our results suggest that MsmSir2 is a novel interaction partner of Ku in prokaryotic cells and may be involved in NHEJ in mycobacteria.

### Sir2 interacts directly with Ku and LigD

**Figure 3:**
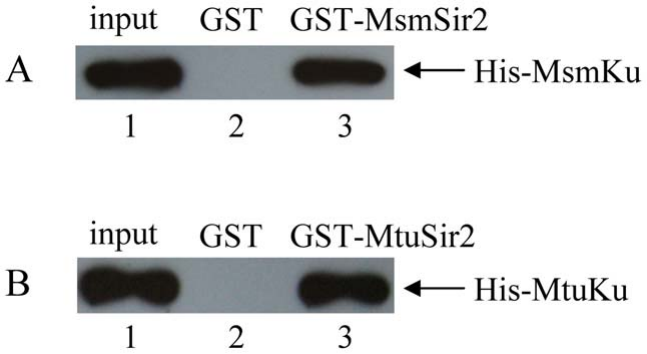
Use of GST pull-down to confirm that Sir2 interacts with Ku. (A) GST pull-down assay. Glutathione sepharose beads were incubated with 2 µg of GST-MsmSir2 (lane 3) or GST (lane 2), followed by incubation with 0.2 µg of His-MsmKu. The bound proteins were probed with an anti-His-tag antibody. Lane 1 contains 20 ng (10% of the total input) of His-MsmKu. (B) GST pull-down assay was also conducted on Sir2 and Ku from *M. tuberculosis*. Glutathione sepharose beads bound with GST-MtuSir2 (lane 3) or GST (lane 2) were incubated with His-MtuKu. The bound proteins separated by SDS-PAGE were analyzed by Western blotting using an anti-His-tag antibody to detect His-tagged MtuKu. Lane 1 contains 20 ng (10% of the total input) of His-MtuKu.

To further investigate the interaction between Sir2 and Ku, an *in vitro* GST pull-down assay was performed. A bacterially-expressed GST-MsmSir2 fusion protein was immobilized on glutathione beads and incubated with purified His-MsmKu protein. Western blotting indicated that Sir2 interacts directly with Ku (Figure 3A). Thus, the interaction observed with the TAP method can also be reconstituted *in vitro*. That Sir2 also interacts directly with Ku from *M. tuberculosis* was confirmed by the same strategy (Figure 3B).

**Figure 4:**
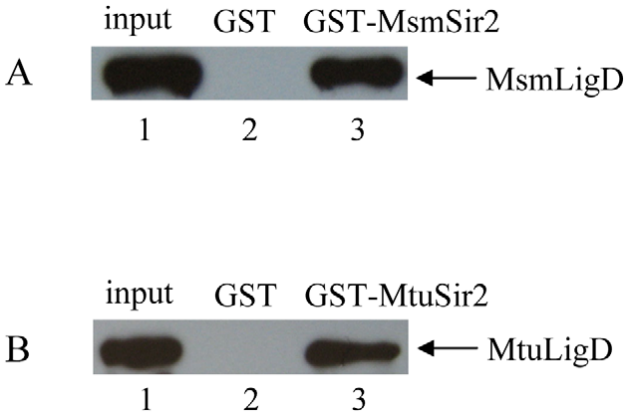
*In vitro* interactions between Sir2 and LigD detected by the GST pull-down assay. (A–B) GST pull-down was used to identify the interaction between Sir2 and LigD in *M. smegmatis* and *M. tuberculosis*. (A) Glutathione sepharose beads were incubated with 2 µg of GST-MsmSir2 (lane 3) or GST (lane 2), followed by incubation with 0.2 µg of His-MsmLigD. The bound proteins were probed with an anti-His-tag antibody. Lane 1 contains 20 ng (10% of the total input) of His-MsmLigD. (B) Glutathione sepharose beads were incubated with 2 µg of GST-MtuSir2 (lane 3) or GST (lane 2), followed by incubation with 0.2 µg of His-MtuLigD. The bound proteins were probed with an anti-His-tag antibody. Lane 1 contains 20 ng (10% of the total input) of His-MtuLigD.

Since the core of the prokaryotic NHEJ apparatus consists of Ku and LigD in interaction with each other, the interaction between Sir2 and LigD must be addressed when determining the function of Sir2 in the NHEJ. As shown in Figure 4, the interaction between Sir2 and LigD protein was confirmed by GST pull-down.

### Sir2 is involved in the mycobacterial NHEJ pathway

**Figure 5:**
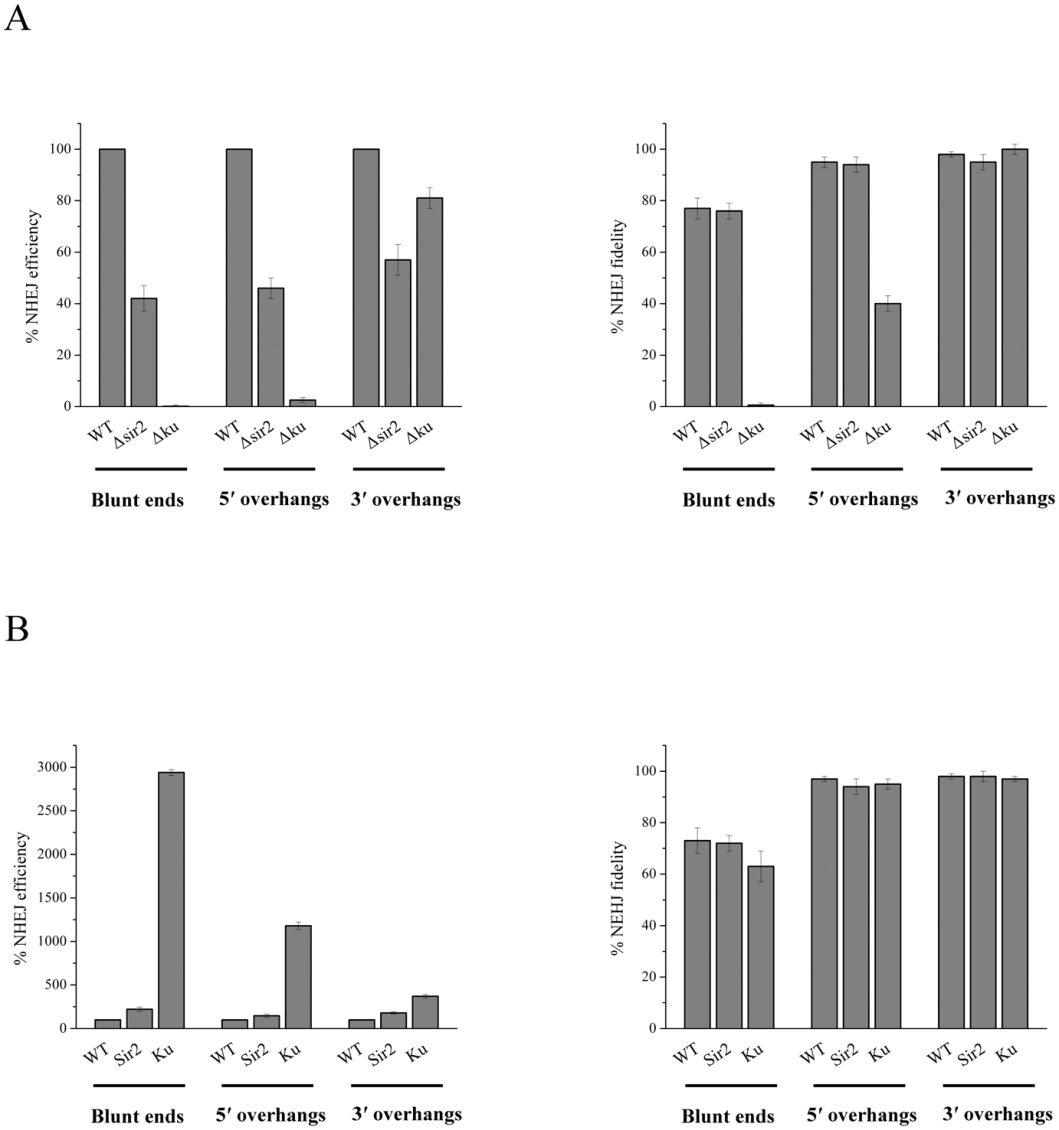
Sir2 is involved in the mycobacterial NHEJ pathway. (A) The *sir2*-deficient strain had reduced NHEJ activity. The *sir2* deletion strains were transformed linear plasmids with three types (blunt, 5′ overhang and 3′ overhang) of DSBs. NHEJ efficiency and fidelity were calculated as described in the materials and methods (*P*-value for statistical significance is less than 0.01). (B) Overexpression of Sir2 enhanced NHEJ activity. Using a similar *in vivo* plasmid-based NHEJ assay, strains overexpressing Sir2 or Ku were transformed with the above three types of linear plasmids (*P*-value<0.05).

As mentioned above, Sir2 interacts with both Ku and LigD in mycobacteria, suggesting its involvement in the mycobacterial NHEJ pathway. A plasmid-based *in vivo* end-joining assay was applied to study the NHEJ pathway in a *sir2*-deficient strain (Figure S5 and S6). The *ku*-deleted strain (Figure S5 and S6) was constructed as a control to evaluate this NHEJ assay method. These mutant strains were generated with a mycobacterial recombineering system [23]. As shown in Figure 5A, the *ku* deletion strain exhibited different deficiencies in the repair of the three DSB end structures (*P*-value<0.01), consistent with previous reports [24]. The overall efficiency of end-joining in the Δ*sir2* mutant was two-fold lower compared with the wild-type strain while the fidelity of NHEJ between the Δ*sir2* mutant and the wild-type strain had no apparent difference, suggesting the involvement of Sir2 in prokaryotic NHEJ efficiency.

**Figure 6:**
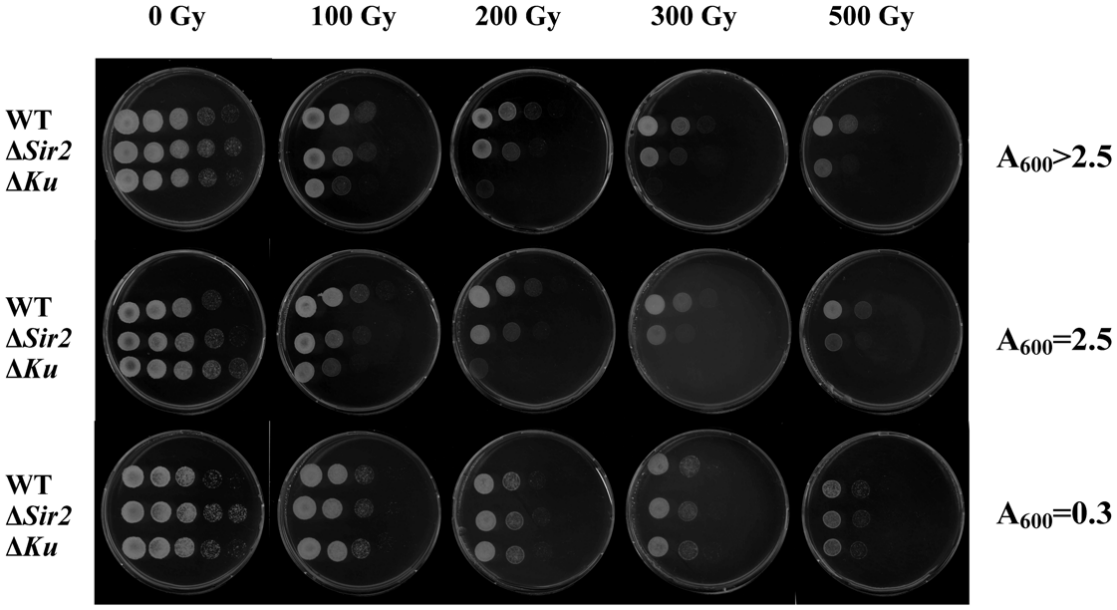
IR sensitivity of sir2- or ku-deficient strains. Cultures of wild-type, Δ*sir2* and Δ*ku* strains of *M. smegmatis* were harvested in the log phase (A_600_ = 0.3), stationary phase (A_600_ = 2.5) or late stationary phase (A_600_>2.5). After irradiation using a ^60^Co source at a dose rate of 14 Gy/min, serial dilutions (10^−1^–10^−4^) of cultures were spotted (20 µl) onto 7H10 plates. The plates were incubated at 37°C for 3 days. Results presented are representative of three replicate experiments.

**Figure 7:**
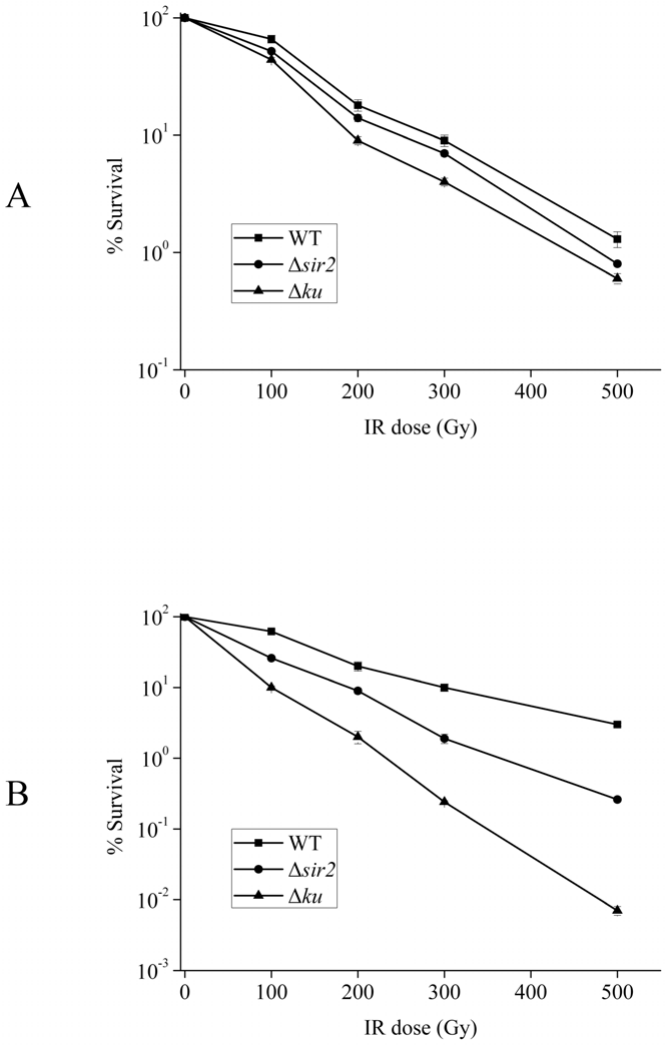
Cell survival after ionizing radiation. Cells in the log or stationary phases were exposed to γ-irradiation at the doses indicated. Cell survival was measured as described in the Materials and methods. (A) Log phase cultures of wild-type, Δ*sir2* and Δ*ku* strains of *M. smegmatis* were sensitive to ionizing radiation to a similar extent. (B) Sensitivity of wild-type, Δ*sir2* and Δ*ku* stationary phase cultures to ionizing radiation. Δ*sir2* and Δ*ku* strains were more sensitive to ionizing radiation than the wild-type (*P*-value<0.05).

In order to further test the role of Sir2 protein in mycobacterial NHEJ, we explored the effect of IR on wild type, *sir2*- or *ku*-deficient strains. As expected, *ku*-deficient cells were extremely sensitive to IR in the stationary phase (Figure 6), consistent with Ku's role in NHEJ. Moreover, *sir2*-deficient cells also presented marked sensitivity to IR in the stationary phase (Figure 6), confirming that Sir2 is involved in the NHEJ pathway. Furthermore, the survival curves (Figure 7) show that irradiated Δ*sir2* stationary phase cells had about a 10-fold reduction in viability compared to wild-type cells, further confirming that Sir2 plays a role in bacterial NHEJ. In addition, the viability of wild-type and *sir2*- or *ku*-deficient strains was affected to a similar extent when exposed to irradiation during the logarithmic phase (Figure 7). It is known that NHEJ is required for prokaryotic DSB repair in the stationary phase [17], [25]; the above evidence that the Δ*sir2* strain was more sensitive to IR in the stationary phase than in the log phase when compared to the wild-type further indicates that Sir2 protein plays a role in NHEJ. Taken together, these results therefore provide strong evidence supporting the hypothesis that Sir2, like Ku, is involved in the mycobacterial NHEJ pathway during the stationary phase.

### Overexpression of Sir2 protein stimulates NHEJ

To further investigate whether overexpression of Sir2 had any influence on NHEJ, the wild-type mc^2^155 carrying a pJRL-Sir2 overexpression plasmid, and control cells with an empty pJRLC vector, were electroporated with a linearized hygromycin-resistant NHEJ assay plasmid. Ku protein was also overexpressed by the pJRL-*ku* plasmid. The influence of Ku and Sir2 on NHEJ activity was analyzed by counting colony numbers and recording colony color after incubation for 3 d at 37°C. As shown in Figure 5B, the efficiency of repairing blunt, 5′ overhang, and 3′ overhang DSBs all increased two-fold in Sir2-overexpressing cells compared with that in control cells, which once again demonstrated the significant role that Sir2 plays in the end-joining process. NHEJ efficiency was enhanced dramatically when Ku was overexpressed, showing its essential role in the prokaryotic NHEJ pathway. Nonetheless, the overall fidelity of the Sir2- or the Ku-overexpressing strain was not affected.

**Figure 8:**
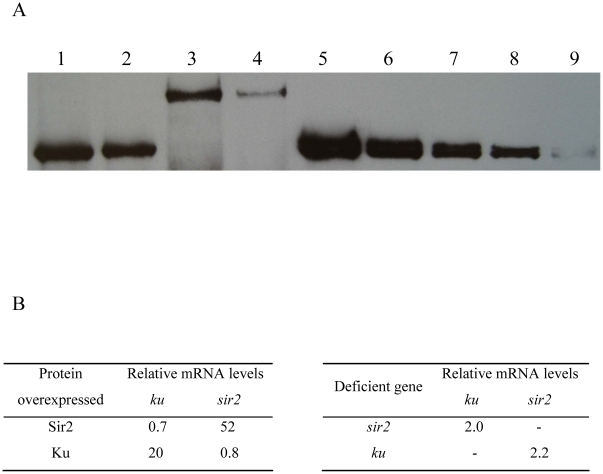
Overexpression of Sir2 reduced the Ku protein level independently of its mRNA level. (A) Western blotting of the protein level of Sir2 (or Ku) after the overexpression of Ku (or Sir2). Lane 1, Sir2-TAP-tagged strain; lane 2, Sir2-TAP-tagged strain overexpressing Ku; lane 3, Ku-TAP-tagged strain; lane 4, Ku-TAP-tagged strain overexpressing Sir2; lanes 5–9 contains 15, 10, 5, 1 and 0.1 µM purified TAP-Sir2 protein, separately. After 3 days incubation at 37°C, the cells were harvested and cell concentration was calculated by measuring the absorbance of the culture at UV 600 nm. Cell lysates prepared from the same cell number were loaded onto the SDS-PAGE gel. Meanwhile, purified TAP-Sir2 that loaded onto the gel with a linear concentration gradient was used as control for the semi-quantitative analysis of Sir2 (or Ku) protein expression. (B) Real-time PCR analysis of the *ku* (or *sir2*) mRNA level when Sir2 (or ku) was overexpressed (left panel) or deleted (right panel). The quantitative data are the ratio of mRNA expression of one gene (*ku* or *sir2*) when the other one was overexpression or deleted relative to the wild-type cells after normalization against the 16S rRNA (*rrsA*) gene.

We next used Western blotting to examine whether the effect of overexpressed Sir2 on NHEJ was correlated with its regulation of Ku protein expression. The TAP-tag knock-in Ku and Sir2 strains were transformed with pJRL-Sir2 and pJRL-Ku, respectively, and the empty pJRLC vector was transformed into each TAP-tagged strain as a control. An anti-ProtA antibody was used in Western blotting analysis to detect TAP-tagged Ku or Sir2. As shown in Figure 8A, there were no apparent differences in the Sir2 protein level between control cells and Ku-overexpressing cells. In contrast, overexpression of Sir2 protein reduced the amount of Ku protein. These results suggest that Sir2 expression downregulated Ku expression.

We also used quantitative real-time PCR to analyze Sir2 and Ku mRNA levels. The Sir2 (or Ku) mRNA levels in wild-type cells did not change significantly after the overexpression of Ku (or Sir2) (Figure 8B). These results suggest that Ku does not participate in the regulation of Sir2 at the mRNA level, and *vice versa*.

## Discussion

The eukaryotic NHEJ system has been extensively studied and has a growing list of new components. In recent years, emerging research on the prokaryotic NHEJ system is focusing on the DNA binding protein Ku and the multifunctional protein LigD, but additional factors involved in the end-joining process remain obscure. Using the yeast two-hybrid system, Sinha et al [18] identified UvrD1, a novel protein interacting with Ku, shedding light on the bacterial NHEJ mechanism by identifying the involvement of alternative proteins. As *M. tuberculosis* spends prolonged periods in non-replicating states within macrophages, the NHEJ system is thought to be an important pathway for its survival in the human body. A deeper understanding of the mycobacterial NHEJ mechanism will help in developing strategies for combatting this pathogen.

This is the first time that the TAP system, a powerful protein purification technique, has been applied in *M. smegmatis*. Using this method, a NAD-dependent deacetylase (MSMEG_5175) named MsmSir2 was identified as a novel Ku binding protein that is involved in the mycobacterial NHEJ system. MsmSir2 has high similarity with eukaryotic Sir2. It has been reported that the Sir2/3/4 complex interacts physically with the NHEJ proteins Yku70 [26] and Yku80 [27] *in vivo*, and that Sir4p plays a major role in the initiation of the interaction process and recruits Sir2p and Sir3p [28]. Interestingly, we found that MsmSir2 interacts directly with MsmKu and MsmLigD *in vitro*. All the above interactions were also observed between Sir2 and Ku and LigD from *M. tuberculosis*. Therefore, our results suggest that Sir2 forms a ternary complex between Ku and LigD, implying that Sir2 plays an important biological role in NHEJ.

In yeast cells, Sir2 has been reported to take part indirectly in the eukaryotic NHEJ pathway, and affects end-joining efficiency by regulating expression of mating-type genes (*HMR*a and *HML*α) [29], [30]. Mutations in *SIR* genes cause the haploid a- or α- mating type cells to have the nonmating phenotype of a/α diploids. Sir^−^ haploid cells lacking mating-type genes have the same NHEJ efficiency as Sir^+^ strains. However, the silent mating-type genes *HML*α and *HMR*a are not present in mycobacteria and the function of Sir2 may not be completely the same from bacteria to human despite highly conserved. Thus MsmSir2 possibly affects bacterial NHEJ by an alternative mechanism. Results from our plasmid-based *in vivo* NHEJ assay clearly suggest the existence of a NHEJ pathway in *M. smegmatis* involving Sir2, but suggest that the role of Sir2 in NHEJ is not as crucial as that of Ku. A probable explanation for this is that the DSBs of the DNA substrate were the complementary sequences generated by restriction enzyme digestion and could be readily ligated. Thus Ku and LigD were sufficient to repair these linear plasmids. However, Sir2 probably accelerates the repair process by facilitating the recruitment of Ku and LigD at the DSB, since a lower level of Ku protein was required for efficient NHEJ when Sir2 was overexpressed. In order to produce more DNA DSBs *in vivo*, IR was utilized for further verification of the role of Sir2 in the NHEJ pathway. IR analysis revealed that the Δ*sir2* strain was around 10-fold more sensitive to IR in the stationary phase than the wild-type strain, suggesting that it has a role in DNA DSB repair after severe DNA damage to cells. Given the ternary complex of Sir2/Ku/LigD and the reduction in NHEJ efficiency in the *sir2*-deficient strain, Sir2 could be a regulator which serves as a scaffold for recruiting Ku and LigD to promote NHEJ activity.

In this study, depletion or overexpression of Sir2 affected NHEJ efficiency, suggesting that Sir2 plays a role in mycobacterial NHEJ. Interestingly, overexpression of Sir2 causes a low level of Ku protein, indicating that there is an elaborate regulation or feedback mechanism that balances the expression of particular proteins in the NHEJ machinery. Although the overexpression of Sir2 seems to be able to compensate for the reduced expression of Ku protein in NHEJ, the amount of Ku mRNA was not affected by the overexpression of Sir2. Likewise the mRNA level of Sir2 was not affected by the overexpression of Ku. Thus, regulation of the amount of these proteins appears to take place during protein translation. These regulatory mechanisms require further investigation.

## Materials and Methods

### Strains and culture conditions

The *M. smegmatis* mc^2^155 strain and its derivatives were grown at 37°C on ADC–containing Middlebrook 7H10 medium (Difco). Strains grown in liquid medium were shaken in Middlebrook 7H9 broth (Difco) supplemented with ADC enrichment media (Difco). When required, 100 µg/ml hygromycin B (Roche Diagnostics) or 20 µg/ml kanamycin (Sigma) was used.

### Construction of plasmids

The coding sequences of Sir2 in *M. tuberculosis* and *M. smegmatis* were amplified by PCR and then cloned into the pGEX-6P-1 vector at the BamHI-XhoI site to generate the fusion plasmids pGEX-6P-1-MtuSir2 and pGEX-6P-1-MsmSir2, respectively. Similarly, the open reading frames encoding Ku and LigD were cloned respectively into pQE30 and pET28a vectors at different sites (Table 1), generating the pQE30-MtuKu and pQE30-MsmKu Ku expression plasmids and the pET28a-MtuLigD and pET28a-MsmLigD LigD expression plasmids. Genomic DNA was isolated and purified with a Genomic DNA Isolation Kit (Omega) according to the manufacturer's instructions. All the plasmids were extracted using a Plasmid DNA Kit (Omega) according to the protocol provided. All the above plasmids were used in the pull-down assay.

**Table 1:** Primers used in plasmid construction.

Plasmid	Primer sequence (5′-3′)	Restriction site
pQE30-MsmKu	TTTTGGATCCATGAACCGTGCGGTACGCCATA	BamHI
pQE30-MsmKu	AAAAAAGCTTCTACGACTTCTTCGCAGCTG	HindIII
pQE30-MtuKu	TTTTGGATCCATGCGAGCCATTTGGAC	BamHI
pQE30-MtuKu	AAAAAAGCTTTCACGGAGGCGTTGGGACGT	HindIII
pGEX-6P-1-MsmSir2	CGCTGGATCCATGCAAGTTACTGTGCTCA	BamHI
pGEX-6P-1-MsmSir2	TTAACTCGAGTCAGGCCGAGCGGTTGAG	XhoI
pGEX-6P-1-MtuSir2	TAAAGGATCCATGCGAGTGGCGGTGCTCA	BamHI
pGEX-6P-1-MtuSir2	TATACTCGAGCTATTTCAGCAGGGCGGGCA	XhoI
pET28a-MsmLigD	TATAGGATCCATGGAGCGCTATGAGCGGGTT	BamHI
pET28a-MsmLigD	GTCGCAAGCTTCTATTCCCACACAACCTCATC	HindIII
pET28a-MtuLigD	ATTAGGATCCATGGGTTCGGCGTCGGAGCAACG	BamHI
pET28a-MtuLigD	TCGAGAAGCTTTCATTCGCGCACCACCTCACTG	HindIII

For the *in vivo* plasmid-based NHEJ assay, *lacZ* reporter plasmids were constructed according to Aniukwu *et al* [24] with slight modifications. Briefly, the promoter of Rv2642 was first amplified from genomic DNA with PCR primers tagged with KpnI and XbaI restriction sites located 5′ and 3′ to the gene, respectively. The amplified fragment was then cloned into the pJV53 vector (a gift from Dr Graham F. Hatfull, University of Pittsburgh, USA), generating pJVR. Subsequently, the *lacZ* gene encoding β-galactosidase was amplified from genomic DNA of *E.coli* strain AB1157 (kindly provided by the Coli Genetic Stock Center, Yale University) and cloned into the pJVR vector using XbaI and NheI restriction sites engineered into the primers. A PstI restriction enzyme site was introduced into the forward primer and the plasmid was named pJRL. As plasmid pJRL contains an intact *lacZ* gene, it was first linearized with a suitable restriction enzyme before NHEJ analysis, since uncut circular plasmids containing functional *lacZ* genes would affect the analysis of NHEJ fidelity. In order to avoid this problem and improve the accuracy of the analysis of NHEJ fidelity, a foreign DNA fragment from combination of the part of ssb and ligD gene of *M. smegmatis* with the HindIII site at the junction was inserted into the *lacZ* gene. This 1.6-kbp EcoRV fragment with an EcoRV site engineered into the primers in each end of the fragment was inserted into the single EcoRV site of pJRL, generating the pJRLE reporter plasmid. Similarly, the XbaI and PstI fragments were inserted into pJRL at the corresponding sites introduced as described above, generating the pJRLX and pJRLP reporter plasmids, respectively. For overexpression analysis, the hygromycin B gene was amplified from plasmid pSMT3 (kindly provided by Dr. Marcus A. Horwitz, University of California Los Angeles, USA) with PCR primers tagged with SpeI and NheI restriction sites. The amplified fragments were then respectively cloned into the pJRL, pJRLE, pJRLX and pJRLP plasmids, generating the pJRLH, pJRLHE, pJRLHX and pJRLHP plasmids for the NHEJ assay in Ku/Sir2 overexpressing strains.

To construct plasmids for overexpression analysis, *sir2* was PCR-amplified and cloned into the pJVR vector using XbaI and NheI restriction enzyme sites engineered into the primers to generate pJVR-Sir2. pJVR-Ku and pJVR-LigD were constructed in a similar manner to overexpress Ku and LigD, respectively. All cloning products were verified by sequencing.

### Construction of the TAP-tag knock-in cassette and its insertion into the C-terminus of ku

The TAP (protA/CBP) tag (kindly provided by Dr. Jikai Wen, University of Birmingham, UK) consists of two immunoglobulin-binding domains of protein A, a cleavage site for the tobacco etch virus (TEV) protease, and the calmodulin-binding peptide (CBP). A knock-in cassette was generated by splicing the TAP-tag gene upstream of the selectable marker gene using overlap extension PCR. The 500 bp upstream and downstream sequences of the *ku* gene at the 3′ termini site termed kua (3′ end of *ku*) and kub (3′ UTR of *ku*) were then amplified by PCR. The kua and kub fragments were inserted adjacent to the N-terminus and C-terminus respectively by overlap extension PCR as described above, to form the final linear targeting substrate (Figure 1). The primers used to generate the Knock-in cassette are shown in Table S1. Subsequently, we used recombineering methods [23] to knock-in the TAP tag flanking the C-terminus of *ku*. Briefly, the linear substrate was introduced into cells containing the recombineering plasmid pJV53 by electroporation, and the cells were allowed to recover for 4 h while shaking at 37°C before being plated onto 7H10 agar. The *ku*-TAP fusion gene was examined by PCR (Table S2) and cells expressing Ku-TAP-tag fusions were validated by Western blotting. The TAP-tag gene was cloned into the plasmid pSMT3 at the BamHI-HindIII site to generate the fusion plasmid expressing TAP-tag protein as a control for Western blotting.

### Tandem affinity purification

A 2 L culture of *M. smegmatis* cells was grown at 37°C for 3 days. After harvesting, cell pellets were washed once with lysis buffer (10 mM Tris-HCl pH 7.9, 0.15 M NaCl, 10% (v/v) glycerol, 0.2% (v/v) Nonidet P-40, 1 mM PMSF) and pelleted again. All subsequent steps were carried out at 4°C. Cell pellets resuspended in lysis buffer were broken up by sonication, and a crude cytoplasmic extract was obtained from the soluble fraction after centrifugation at 16,000 *g* for 1 h. The supernatant was added to a Polyprep column (Bio-Rad, Hercules, CA) with a 200 µl bed volume of IgG-Sepharose 6 Fast Flow beads (Pharmacia) and rotated for 2 h at 4°C. The resin was washed three times with lysis buffer, and twice with TEV buffer (1 mM DTT and 0.5 mM EDTA in lysis buffer). Bound complexes were cleaved from the matrix with 100 units of TEV protease (Invitrogen, Carlsbad, CA) in 1 ml TEV cleavage buffer while rotating the column overnight at 4°C and then recovered by elution. Subsequently, the eluate was diluted with 3 ml of calmodulin binding buffer (10 mM Tris-HCl pH 7.9, 0.15 M NaCl, 1 mM MgCl_2_, 1 mM imidazole, 0.1% (v/v) Nonidet P-40, 10 mM β-mercaptoethanol and 2 mM CaCl_2_) plus 3 µl of 1 M CaCl_2_, and transferred to a Polyprep column containing 0.2 ml calmodulin affinity resin (Pharmacia) equilibrated with calmodulin binding buffer. After incubation for 2 h at 4°C, the matrix was washed four times with 10 ml of calmodulin binding buffer and eluted with 1.5 ml of calmodulin elution buffer (10 mM Tris-HCl pH 7.9, 0.15 M NaCl, 10 mM EGTA). The final eluate was concentrated by acetone precipitation and dissolved with 10 µl sodium dodecyl sulfate (SDS) loading buffer. After boiling, the protein sample was separated by SDS-polyacrylamide gel electrophoresis (SDS-PAGE) and silver stained with a PlusOne silver staining kit (GE Healthcare) according to the manufacturer's protocol.

### Mass spectrometric analysis

Individual protein bands were excised from polyacrylamide gels, digested with trypsin, and analyzed by liquid chromatography-tandem mass spectrometry. MS data were searched using SEQUEST against NCBI *M. smegmatis* protein database and results were filtered and displayed using the Bioworks 3.2.

### Construction of *M. smegmatis sir2* and *ku* null mutants

*M. smegmatis* strain null alleles were constructed by replacing the bulk of the open reading frame of each gene with the hygromycin B resistance gene using a mycobacterial recombineering system [23]. Briefly, the allelic exchange substrates were constructed as described above by overlap extension PCR. The allelic exchange substrate for every ORF was constructed by PCR amplification of approximately 500 bp corresponding to the upstream and downstream regions, and was subsequently inserted adjacent to the hygromycin-resistance gene by overlap extension PCR. The primers used for amplifying the left and right arms of the knock-out substrate for every gene are listed in Table S3. All gene disruptions were tested by PCR using flanking DNA sequences as primers and were further confirmed by DNA sequencing of the PCR products and Southern blotting. The primers used for the PCR analysis are listed in Table S4.

### Southern blotting

To confirm the constructed *sir2* and *ku* mutants, genomic DNA was isolated from M. semgmatis wild-type and *sir2* and *ku* mutants using a genomic DNA isolation kit (Omega) and then digested with PstI restriction enzymes. Fragments containing the 5′ flanking region of the *sir2* or *ku* gene, labeled with DIG using a DIG High Prime DNA Labeling Kit (Roche), were used as probes. Southern blotting was performed using a DIG High Prime DNA Detection Kit (Roche) according to the manufacturer's protocol.

### Protein expression and purification

Plasmids used for pull-down assays were all transformed into BL21 (DE3) cells. Each transformed clone was cultured in 750 ml LB medium at 37°C until the A_600_ reached 0.5. After diluting to a final concentration of 0.4 mM IPTG, the culture was incubated for a further 10 h at 20°C with constant shaking. Harvested cell pellets were washed once with water and stored at −80°C. The *MtuKu* and *MsmKu* genes were inserted into separate pQE30 vectors and expressed in *E. coli*. The resulting His-tagged proteins were purified using Ni-NTA resin (GE Healthcare). Proteins bound to the resin were equilibrated with 50 ml of binding buffer (20 mM Tris-HCl pH 7.9, 10 mM imidazole and 500 mM NaCl). After washing with washing buffer (20 mM Tris-HCl pH 7.9, 80 mM imidazole and 500 mM NaCl), proteins were eluted with 300 mM imidazole. Similarly, MtuLigD, MsmLigD were purified using the above methods. Purified proteins were dialyzed against dialysis buffer (50 mM Tris-HCl pH 7.5, 200 mM NaCl, and 20% glycerol). After dialysis and concentration, the proteins were stored either short-term at −20°C or long-term at −80°C.

MtuSir2-GST, MsmSir2-GST and GST alone were purified using glutathione-Sepharose 4B beads (GE Healthcare). After washing with buffer A (20 mM Tris-HCl pH 7.5, 500 mM NaCl and 10% glycerol), the proteins were eluted with buffer B (10 mM reduced glutathione, 50 mM Tris-HCl pH 7.5 and 500 mM NaCl). The purified proteins were analyzed by 12% SDS-PAGE. Protein concentration was determined using the Coomassie Bradford method (Thermo Scientific Pierce) with bovine serum albumin as the standard.

### GST pull-down assays

The purified MsmSir2-GST protein (20 µg) or the GST protein itself (20 µg) was incubated with 40 µl of glutathione-Sepharose 4B beads in 500 µl of buffer A (50 mM Tris-HCl pH 7.5, 200 mM NaCl, and 10% glycerol) with rotation at 4°C for 2 h. After washing five times with 1 ml of buffer A containing 0.1% Nonidet P-40, the beads were then incubated with purified Ku protein (20 µg) at 4°C for 8 h. Finally, after extensive washing, all bound protein was eluted by boiling the beads in 40 µl of 2×SDS loading buffer and resolved by 12% SDS-PAGE for subsequent visualization by Western blotting. The immunoblot was probed with a monoclonal antibody directed against the His tag (1∶2000 dilution) and with an anti-GST antibody (1∶2000 dilution). The pull-down assay was used to investigate the other protein-protein interactions in the same way.

The protein-protein interactions between LigD and Sir2 were also analyzed using the GST pull-down assay described above.

### Preparation of substrate DNA

The plasmids (pJRL, pJRLE, pJRLX and pJRLP) used in the *in vivo* NHEJ assay were constructed according to Aniukwu *et al* [24] with slight modifications. Briefly, the digestion sites designed to generate defined double strand breaks were XbaI (5′ overhangs), PstI (3′ overhangs), and EcoRV (blunt end). The plasmids (pJRLH, pJRLHE, pJRLHX and pJRLHP) for the NHEJ assay in Sir2/Ku-overexpressed strains were hygromycin resistant instead of kanamycin resistant.

To generate the linear DNA substrates with different end, the plasmids above were digested with appropriate enzyme (XbaI, PstI or EcoRV), and then digested with HindIII that cuts within the foreign DNA fragment. The linearized fragments were isolated and purified from the gel using a Promega gel-extraction kit. The DNA concentration was determined by UV spectrophotometry (Thermo-spectronic).

### Evaluation of DSB repair efficiency and fidelity using the *in vivo* DNA end-joining assay

To document the role of Sir2 in mycobacterial NHEJ *in vivo*, we employed a plasmid-rejoining assay. The NHEJ assay was performed as described previously [24]. Briefly, cells were electroporated with between 10 and 100 ng linearized plasmid DNA or between 5 and 50 ng uncut circular plasmid and then incubated in 1 ml LB medium at 37°C, and rotated at 170 rpm for 3 h. A portion of the transformation mixtures was then plated in triplicate on LB agar medium containing 20 µg/ml kanamycin and 50 µg/ml X-gal. 100 µg/ml hygromycin was added to the above medium for overexpression assays. Plates were incubated for 3 days at 37°C, and colonies were counted manually. Efficiency and fidelity were calculated according to Aniukwu *et al* [24]. Each experiment was repeated at least five times.

### Ionizing radiation (IR) assays

Irradiation was carried out using a ^60^Co source at a dose rate of 14 Gy/min. Cultures were collected in the log phase, stationary phase, or late-stationary phase (corresponding to the different A_600_ units indicated in Figure 6). Following 100, 200, 300 and 500 Gy irradiation, aliquots of 10-fold gradient dilutions were spotted onto 7H10 Middlebrook agar (Difco). In a separate experiment serially diluted samples from the log phase or stationary phase were plated on 7H10 Middlebrook agar (Difco), and colonies were counted after 3 days incubation at 37°C. Non-irradiated samples of each cell type were used as controls in both experiments. Each experiment was repeated three times.

### Western blotting

Western blotting was used for detecting TAP protein expression; proteins were electrophoresed on 12% SDS–polyacrylamide gels and transferred by electroblotting to 0.2 mm PVDF membranes (GE, Healthcare). Hybridizations with antibodies were performed according to the manufacturer's recommendations. The primary antibody used was a peroxidase anti-peroxidase complex (1∶2000, catalogue number P1291; Sigma-Aldrich) for detecting the protA tag. Bound primary antibody was detected and visualized via incubation with a secondary HRP-linked anti rabbit IgG (1∶5000, GE, Healthcare) and chemiluminescent substrate (ECL-plus substrate, GE, Healthcare). Western blotting in the GST pull-down assays was similar to that described above except that the primary antibody was an anti-his-tag antibody and the secondary antibody was an HRP-linked anti-mouse IgG.

### RNA extraction

RNA was isolated from 20 ml cultures of wild-type *M. smegmatis* mc^2^155, Δ*sir2* and Δ*ku* mutant strains, and *M. smegmatis* mc^2^155 carrying different plasmids (pJRL-Sir2, pJRL-Ku, pJRLC). Bacteria were pelleted, then resuspended in 1 ml of lysis buffer and placed in a Lysing Matrix A tube. Lysis was performed in a FastPrep-24 instrument for 45 s at 6500 rpm. The aqueous phase was extracted with 200 µl chloroform, and the RNA was precipitated with isopropanol. After washing in 70% ethyl alcohol, the RNA was eluted in 40 µl of RNase-free water. Chromosomal DNA contamination was removed from the eluted total RNA by treatment with DNase I (MBI Fermentas), followed by heat inactivation of the enzyme. The digested products were then reverse-transcribed with random hexamer primers (MBI Fermentas) to generate cDNA according to the manufacturer's recommendations.

### Quantitative real-time PCR

Quantitative real-time PCR was performed with the SYBR Green PCR kit (Qiagen) on a Rotor-Gene 6000 instrument (Corbett Life Science). 20 µl reactions were set up according to the standard protocol. Sequences of each primer are given in Table S5. All reactions were performed in triplicate. Reactions were heated to 95°C for 10 min followed by cycling for 45 cycles of 95°C for 15 s, 58°C for 15 s, and 72°C for 15 s. At the end of the PCR, melting curve analysis was performed and PCR products were analyzed on an agarose gel to ensure product specificity. The mRNA expression level for each target gene was normalized to the 16S rRNA gene (*rrsA*) expression level.

## Supporting Information

Figure S1 **Construction of** ***M. smegmatis*** **strains expressing TAP-tagged Ku or Sir2.** Validation of TAP-tagged *ku* and *sir2* by PCR analysis. The *ku* and *sir2* genes containing TAP-tag knock-in cassettes were constructed according to the protocol in Figure 1. The TAP tag was targeted to the C-terminal end of the *ku* or *sir2* locus, and positive strains were validated by PCR. In the left panel, lanes 1 and 2, PCR results using primers *x*kia and *x*kib (lane 1,1.3 kbp TAP-tagged *ku* locus, lane 2, wild-type); lanes 3 and 4, PCR results using primers *x*kia and *x*kic (lane 3, 2.9 kbp TAP-tagged *ku* locus, lane 4, 1.3 kbp wild-type locus), *x* in the top panel is the *ku* gene. A wild-type strain was used as a control. The right panel shows the PCR analysis of *sir2* using its corresponding primers. *x* here represents the *sir2* gene. Lanes 1 and 2, PCR results using primers *x*kia and *x*kib (lane 1,1.5 kbp TAP-tagged *sir2* locus, lane 2, wild-type), lanes 3 and 4, PCR results using primers *x*kia and *x*kic (lane 3, 3 kbp TAP-tagged *sir2* locus, lane 4, 1.3 kbp wild-type locus).
(TIF)

Figure S2 **Western blotting of TAP-tagged Ku and Sir2 protein.** The TAP-tagged fusion protein was validated by Western blotting using an anti-ProtA antibody. Only TAP-tag protein was expressed as a control.
(TIF)

Figure S3 **Identification of Sir2 as a Ku-binding protein.** The Ku-binding partners were obtained by tandem affinity purification of TAP-tagged Ku. Protein complexes were visualized by silver staining after separation by SDS-PAGE. Several specific bands were excised and subjected to mass spectrometry. Lane 1, the TAP tag alone as a control; lane 2, TAP-tagged Ku complex; M, Protein molecular weight marker. This is an independent replication of the TAP experiment.
(TIF)

Figure S4 **Protein sequence analysis of various Sir2 proteins.** (A) Alignment of Sir2 proteins from bacteria to humans. The NAD^+^-binding residues are marked by triangles. (B) Phylogenetic relationship of Sir2 among different species. The phylogenetic tree was constructed by the neighbor-joining method using the MEGA 4.1 software. The species abbreviations and the protein accession numbers are: Sc_Sir2, *S. cerevisiae* Sir2, NP_010242; Mt_Sir2, *M. tuberculosis* Sir2, NP_215667; Ms_Sir2, *M. smegmatis* Sir2, YP_889421; Mm_SIRT5, *M. musculus* SIRT5, NP_849179; Hs_SIRT5, *H. sapiens* SIRT5, NP_112534; Ec_CobB, *E. coli* CobB, NP_415638.
(TIF)

Figure S5 **Construction of the** ***sir2*** **and** ***ku*** **deletion strains.** The *sir2* or *ku* gene was deleted from the *M. smegmatis* genome using the mycobacterial recombineering system. The knock-out cassette was generated by overlap extension PCR, in which the two 500 bp sequences fragments flanking each of the ends of ku were fused with the terminal of the Hyg fragment. After the knock-out cassette was transformed into the strains using the recombineering plasmid pJV53, positive recombinants were identified by PCR analysis. In the left panel, lanes 1 and 2, PCR results when primers *x*koa and *x*kob were used (lane 1, 690 bp *sir2*-deletion, lane 2, wild-type), lane 3 and 4, PCR results with primers *x*koa and *x*koc (lane 3, 2.2 kbp *sir2*-deficient locus, lane 4, 1.9 kbp wild-type locus), *x* in the top panel is the *sir2* gene. A wild-type strain was used as a control. The right panel shows the PCR analysis of the *ku* mutant using corresponding primers. *x* here indicates the *ku* gene. Lanes 1 and 2, PCR results using primers *x*koa and *x*kob (lane 1, 800 bp *ku*-deficient locus, lane 2, wild-type), lanes 3 and 4, PCR results using primers *x*koa and *x*koc (lane 3, 2.4 kbp *ku*-deficient locus, lane 4, 2.2 kbp wild-type locus).
(TIF)

Figure S6 **Southern blot analysis of the** ***sir2*** **and** ***ku*** **deletion strains.** (A) Genomic DNA from *M. smegmatis* wild-type (lane 1) and *sir2* mutant (lane2) strains was digested with PstI and probed with 462-bp gene fragment containing the 5′ flanking region of the *sir2* gene. Southern blot analysis revealed the expected fragment of 1700 bp for wild-tpye and larger than 2000 bp for *sir2* mutant. (B) Genomic DNA from *M. smegmatis* wild-type (lane 1) and *ku* mutant (lane2) strains was digested with PstI and probed with 497-bp gene fragment containing the 5′ flanking region of the *ku* gene. Southern blot analysis revealed the expected fragment of 3200 bp for wild-tpye and larger than 3300 bp for *ku* mutant.
(TIF)

Table S1 **Primers used for generating the TAP-tag knock-in cassette.**
(DOC)

Table S2 **Primers used for PCR analysis of TAP-tag knock-in strains.**
(DOC)

Table S3 **Primers used for generating the** ***sir2*** **or** ***ku*** **knock-out cassettes.**
(DOC)

Table S4 **Primers used for PCR analysis of** ***sir2*** **or** ***ku*** **knock-out strains.**
(DOC)

Table S5 **Primers used in qRT-PCR.**
(DOC)
