# Correction: A Sir2-Like Protein Participates in Mycobacterial NHEJ

**DOI:** 10.1371/annotation/a08b91f7-bf59-4384-8464-fca428ef15ef

**Published:** 2011-07-12

**Authors:** Zhongdao Li, Jikai Wen, Yaning Lin, Shihua Wang, Peng Xue, Zhiping Zhang, Ying Zhou, Xiao Wang, Li Sui, Li-Jun Bi, Xian-En Zhang

Author Zhongdao Li is missing an affiliation. The complete byline and affiliations are:

Zhongdao Li1,2,3, Jikai Wen4, Yaning Lin5, Shihua Wang5, Peng Xue2, Zhiping Zhang1, Ying Zhou2, Xiao Wang6, Li Sui6, Li-Jun Bi2*, Xian-En Zhang1*

1 State Key Laboratory of Virology, Wuhan Institute of Virology, Chinese Academy of Sciences, Wuhan, China,

2 National Laboratory of Biomacromolecules and Proteomics Platform, Institute of Biophysics, Chinese Academy of Sciences, Beijing, China,

3 Graduate School, Chinese Academy of Sciences, Beijing, China,

4 School of Biosciences, University of Birmingham, Edgbaston, Birmingham B15 2TT, UK,

5 College of Life Sciences, Fujian Agriculture and Forestry University, Fuzhou, China,

6 Department of Nuclear Physics, China Institute of Atomic Energy, Beijing, China 

