# Supplementary figures and images for: A Sir2-Like Protein Participates in Mycobacterial NHEJ
Source: PLoS One. 2011 May 26;6(5):e20045. doi: 10.1371/journal.pone.0020045 (PMC3102665; doi:10.1371/journal.pone.0020045)

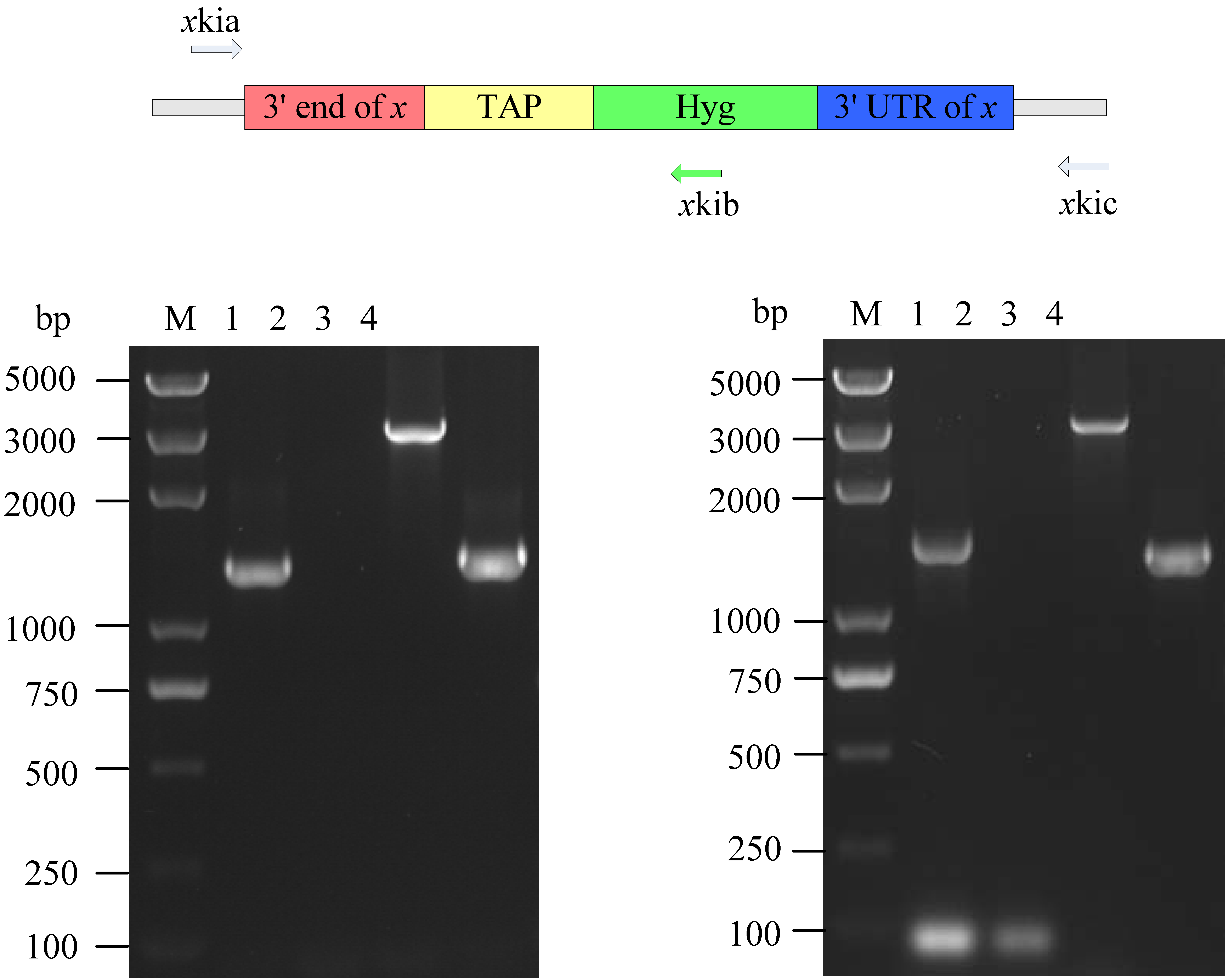

Supplement: Figure S1 — Construction of M. smegmatis strains expressing TAP-tagged Ku or Sir2. Validation of TAP-tagged ku and sir2 by PCR analysis. The ku and sir2 genes containing TAP-tag knock-in cassettes were constructed according to the protocol in Figure 1. The TAP tag was targeted to the C-terminal end of the ku or sir2 locus, and positive strains were validated by PCR. In the left panel, lanes 1 and 2, PCR results using primers xkia and xkib (lane 1,1.3 kbp TAP-tagged ku locus, lane 2, wild-type); lanes 3 and 4, PCR results using primers xkia and xkic (lane 3, 2.9 kbp TAP-tagged ku locus, lane 4, 1.3 kbp wild-type locus), x in the top panel is the ku gene. A wild-type strain was used as a control. The right panel shows the PCR analysis of sir2 using its corresponding primers. x here represents the sir2 gene. Lanes 1 and 2, PCR results using primers xkia and xkib (lane 1,1.5 kbp TAP-tagged sir2 locus, lane 2, wild-type), lanes 3 and 4, PCR results using primers xkia and xkic (lane 3, 3 kbp TAP-tagged sir2 locus, lane 4, 1.3 kbp wild-type locus). (TIF) [file pone.0020045.s001.tif]

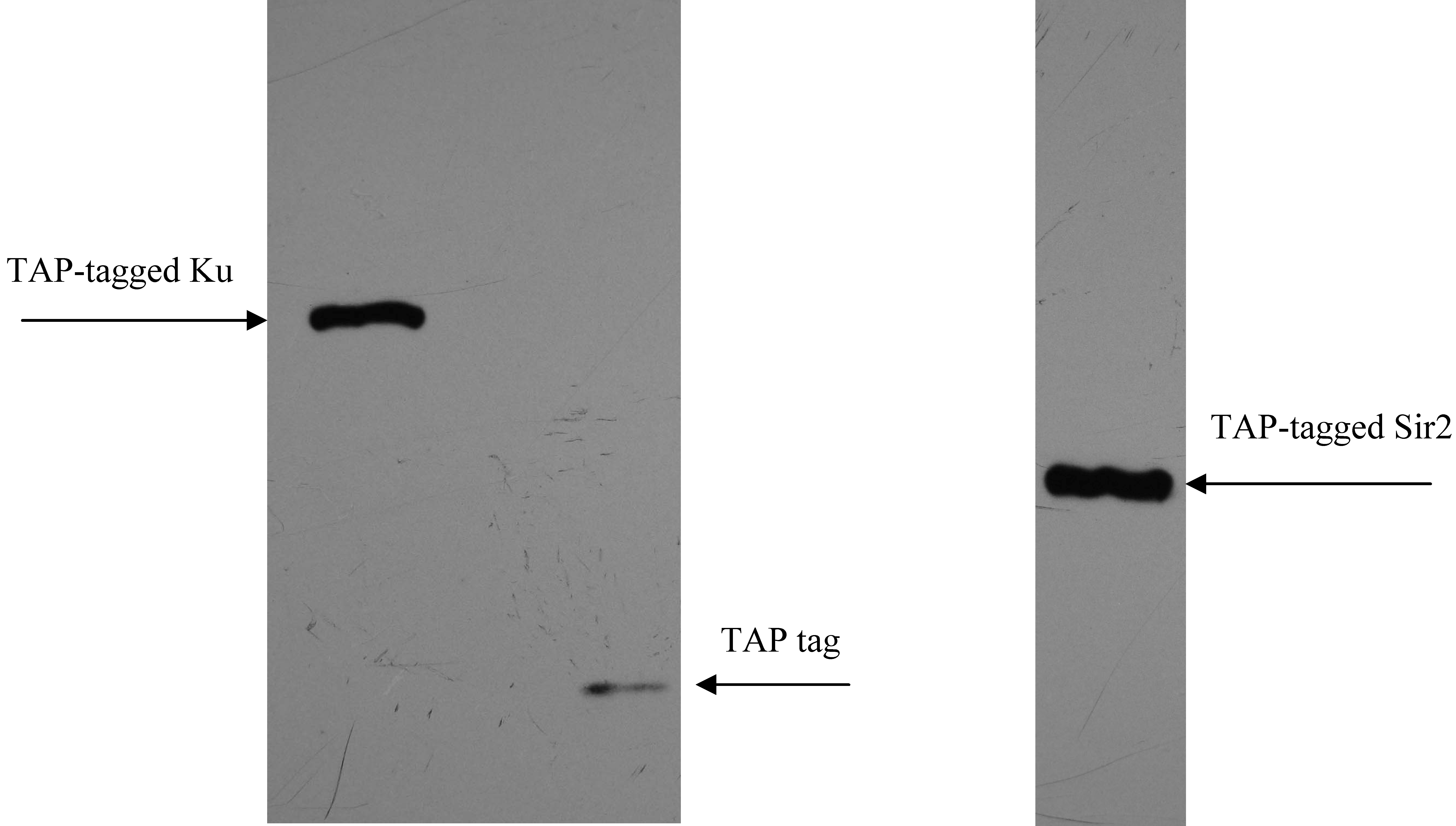

Supplement: Figure S2 — Western blotting of TAP-tagged Ku and Sir2 protein. The TAP-tagged fusion protein was validated by Western blotting using an anti-ProtA antibody. Only TAP-tag protein was expressed as a control. (TIF) [file pone.0020045.s002.tif]

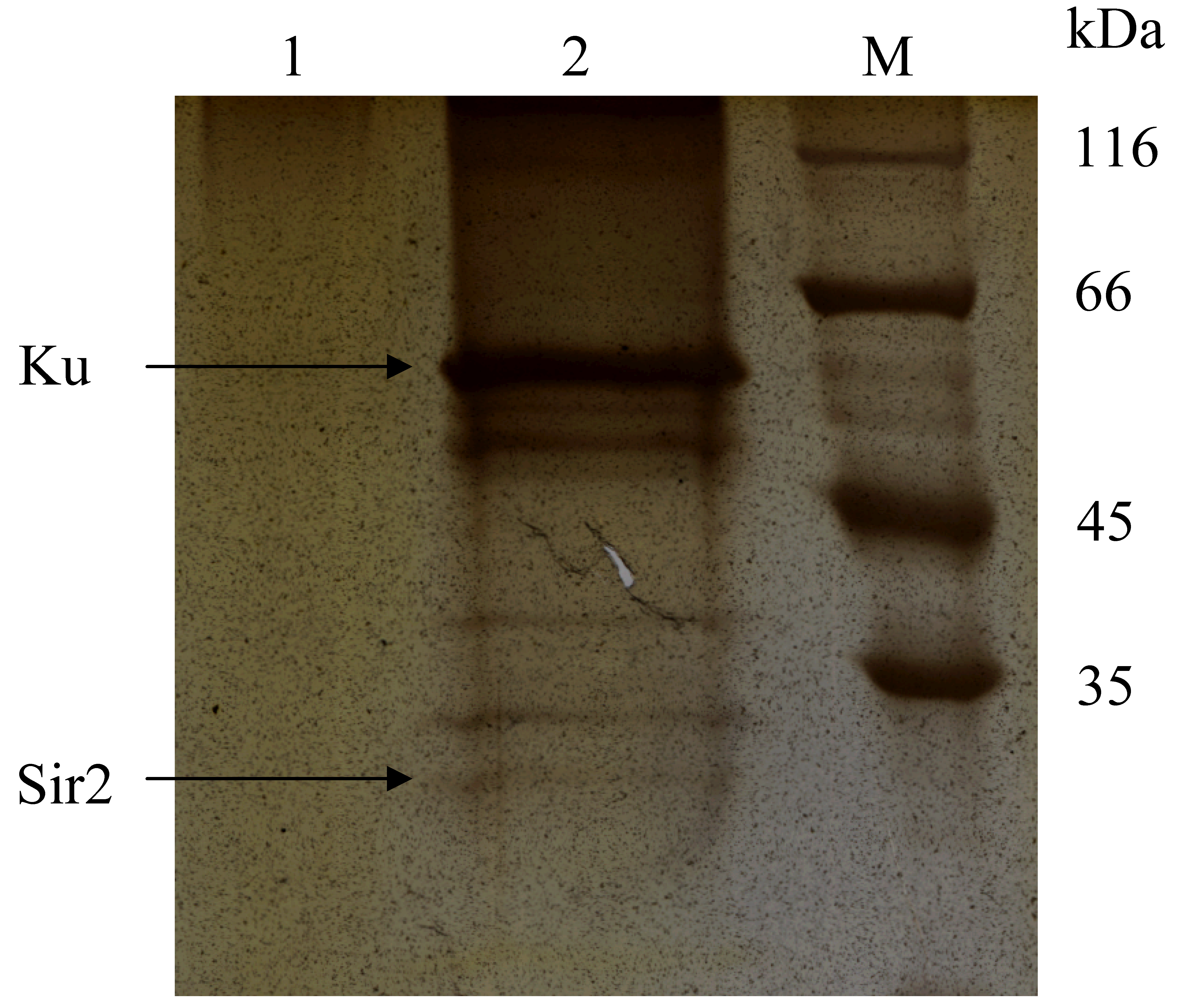

Supplement: Figure S3 — Identification of Sir2 as a Ku-binding protein. The Ku-binding partners were obtained by tandem affinity purification of TAP-tagged Ku. Protein complexes were visualized by silver staining after separation by SDS-PAGE. Several specific bands were excised and subjected to mass spectrometry. Lane 1, the TAP tag alone as a control; lane 2, TAP-tagged Ku complex; M, Protein molecular weight marker. This is an independent replication of the TAP experiment. (TIF) [file pone.0020045.s003.tif]

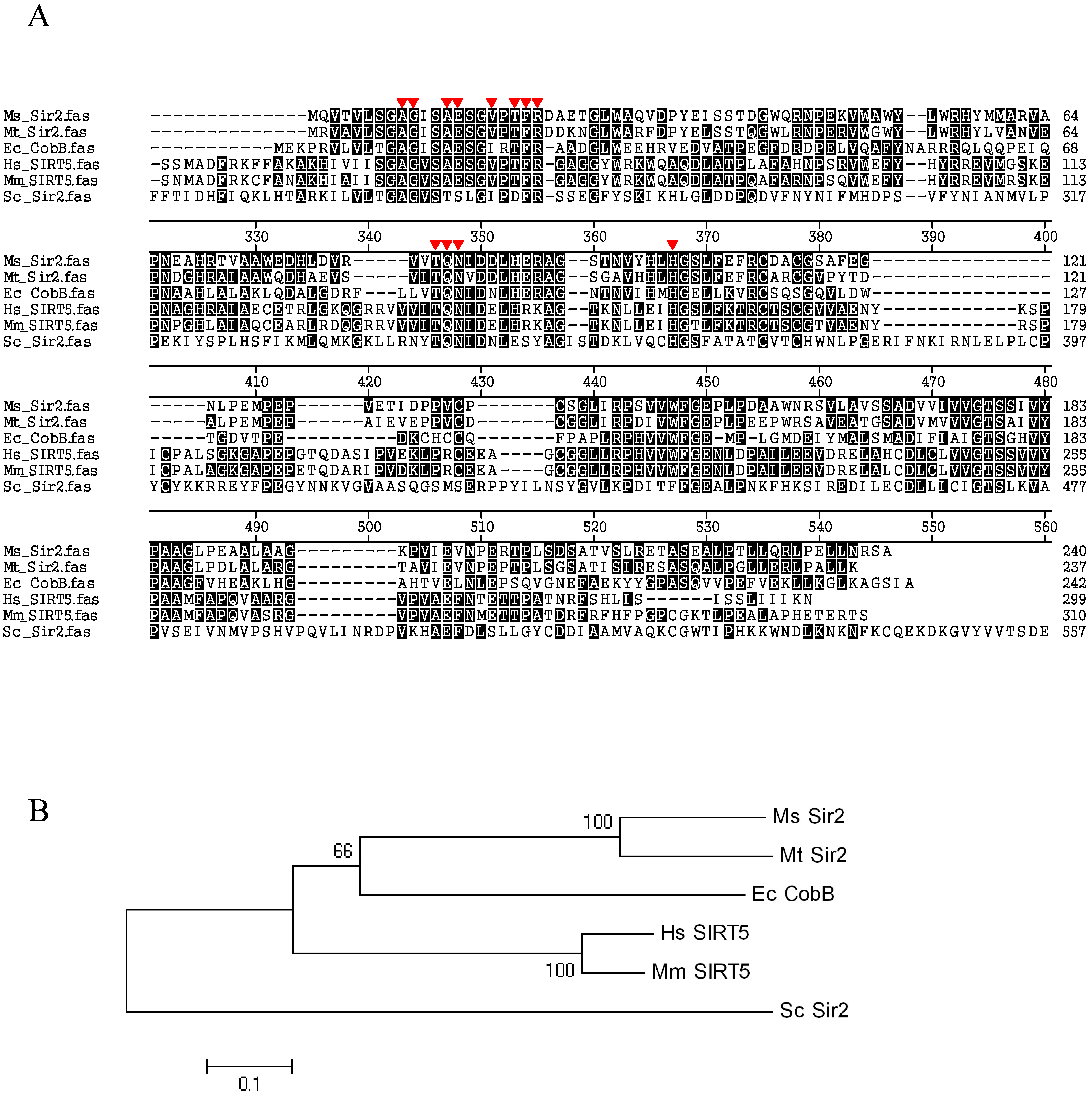

Supplement: Figure S4 — Protein sequence analysis of various Sir2 proteins. (A) Alignment of Sir2 proteins from bacteria to humans. The NAD+-binding residues are marked by triangles. (B) Phylogenetic relationship of Sir2 among different species. The phylogenetic tree was constructed by the neighbor-joining method using the MEGA 4.1 software. The species abbreviations and the protein accession numbers are: Sc_Sir2, S. cerevisiae Sir2, NP_010242; Mt_Sir2, M. tuberculosis Sir2, NP_215667; Ms_Sir2, M. smegmatis Sir2, YP_889421; Mm_SIRT5, M. musculus SIRT5, NP_849179; Hs_SIRT5, H. sapiens SIRT5, NP_112534; Ec_CobB, E. coli CobB, NP_415638. (TIF) [file pone.0020045.s004.tif]

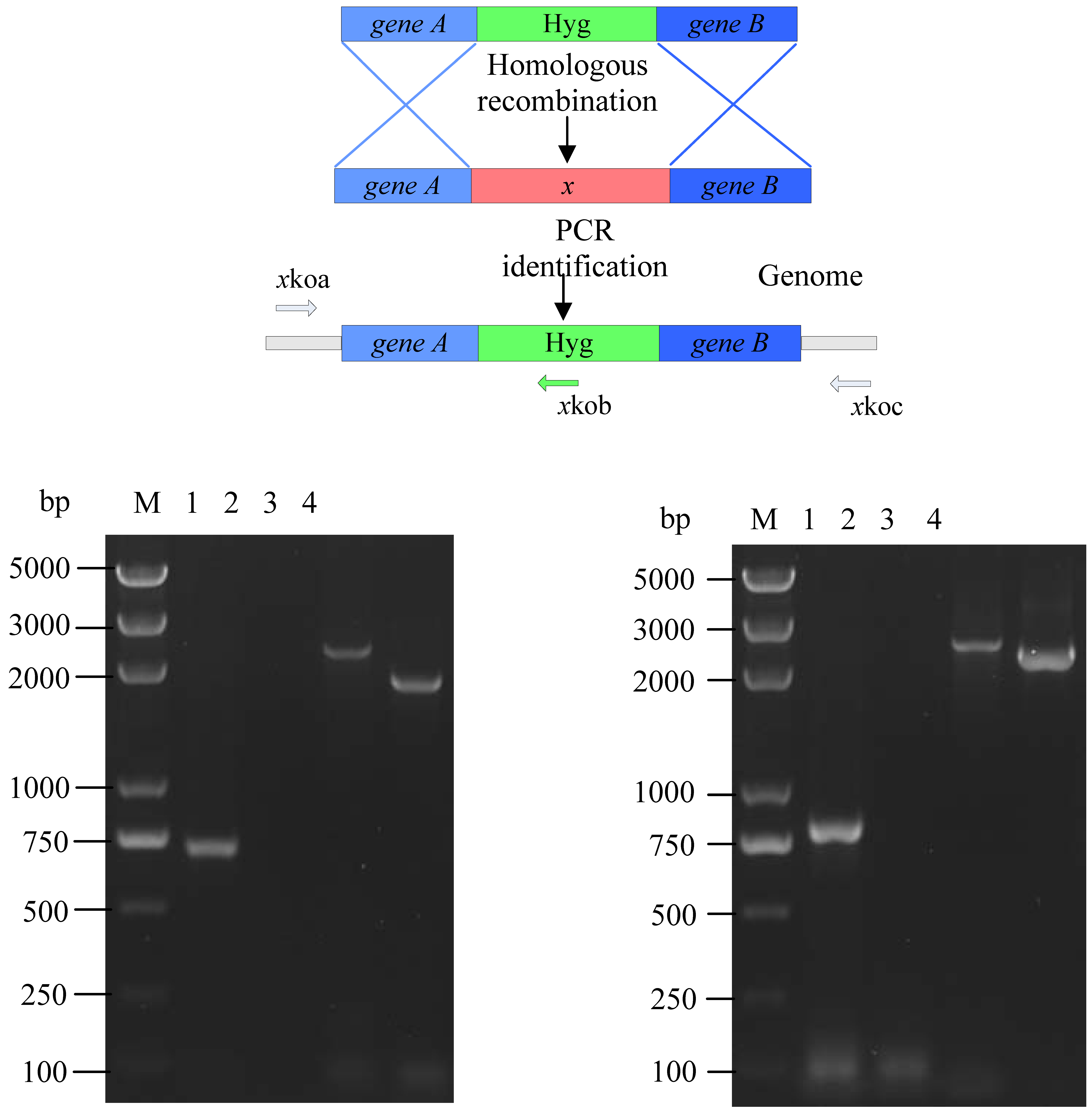

Supplement: Figure S5 — Construction of the sir2 and ku deletion strains. The sir2 or ku gene was deleted from the M. smegmatis genome using the mycobacterial recombineering system. The knock-out cassette was generated by overlap extension PCR, in which the two 500 bp sequences fragments flanking each of the ends of ku were fused with the terminal of the Hyg fragment. After the knock-out cassette was transformed into the strains using the recombineering plasmid pJV53, positive recombinants were identified by PCR analysis. In the left panel, lanes 1 and 2, PCR results when primers xkoa and xkob were used (lane 1, 690 bp sir2-deletion, lane 2, wild-type), lane 3 and 4, PCR results with primers xkoa and xkoc (lane 3, 2.2 kbp sir2-deficient locus, lane 4, 1.9 kbp wild-type locus), x in the top panel is the sir2 gene. A wild-type strain was used as a control. The right panel shows the PCR analysis of the ku mutant using corresponding primers. x here indicates the ku gene. Lanes 1 and 2, PCR results using primers xkoa and xkob (lane 1, 800 bp ku-deficient locus, lane 2, wild-type), lanes 3 and 4, PCR results using primers xkoa and xkoc (lane 3, 2.4 kbp ku-deficient locus, lane 4, 2.2 kbp wild-type locus). (TIF) [file pone.0020045.s005.tif]

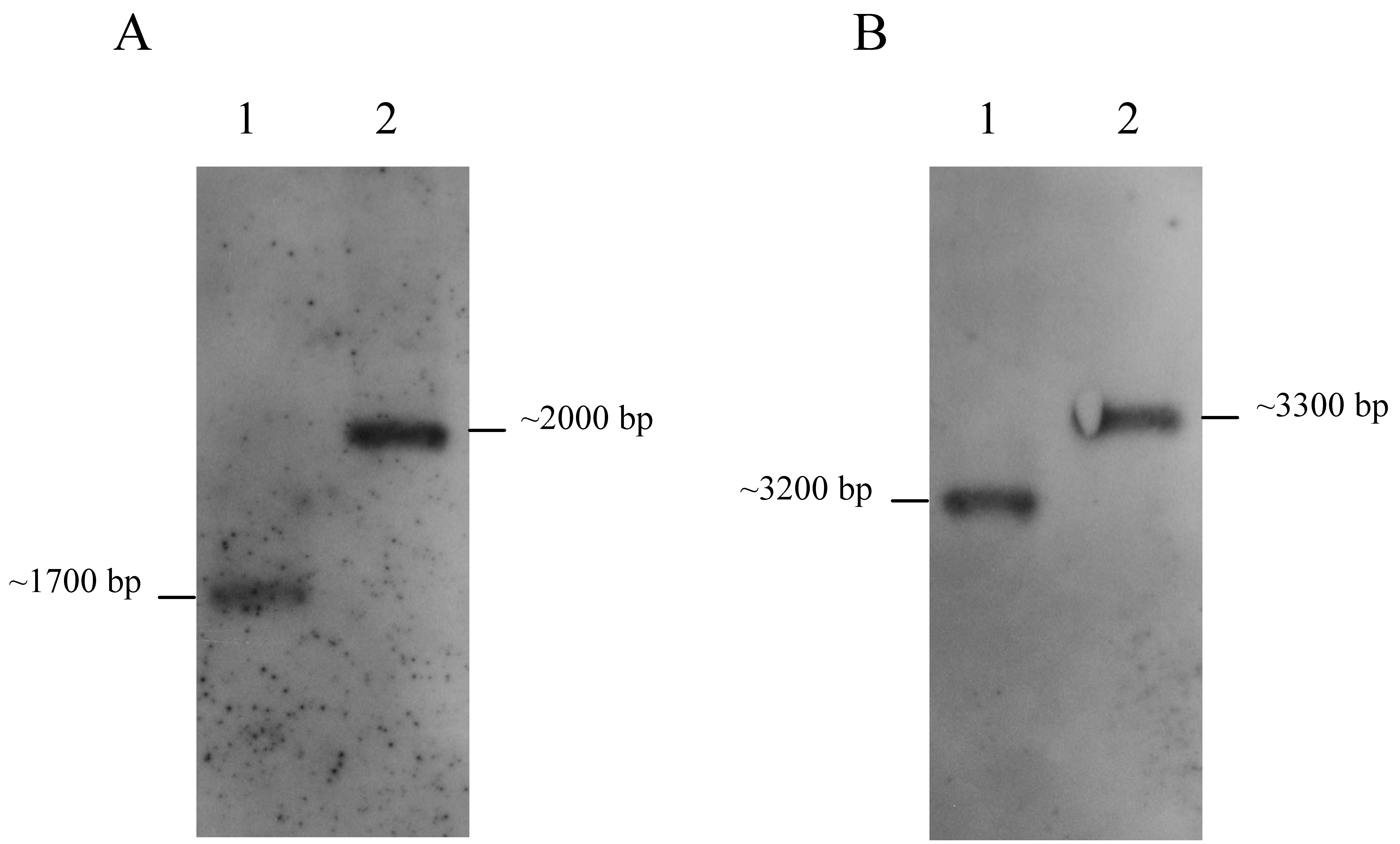

Supplement: Figure S6 — Southern blot analysis of the sir2 and ku deletion strains. (A) Genomic DNA from M. smegmatis wild-type (lane 1) and sir2 mutant (lane2) strains was digested with PstI and probed with 462-bp gene fragment containing the 5′ flanking region of the sir2 gene. Southern blot analysis revealed the expected fragment of 1700 bp for wild-tpye and larger than 2000 bp for sir2 mutant. (B) Genomic DNA from M. smegmatis wild-type (lane 1) and ku mutant (lane2) strains was digested with PstI and probed with 497-bp gene fragment containing the 5′ flanking region of the ku gene. Southern blot analysis revealed the expected fragment of 3200 bp for wild-tpye and larger than 3300 bp for ku mutant. (TIF) [file pone.0020045.s006.tif]
