# Supplementary material for: A Sir2-Like Protein Participates in Mycobacterial NHEJ
Source: PLoS One. 2011 May 26;6(5):e20045. doi: 10.1371/journal.pone.0020045 (PMC3102665; doi:10.1371/journal.pone.0020045)
Supplement: Table S1 — Primers used for generating the TAP-tag knock-in cassette. (DOC) [file pone.0020045.s007.doc]

| **Primer** | **Sequence (5′-3′)** |
| --- | --- |
| **T5** | ATGGAAAAGAGAAGATGGAAAAAGAA |
| **T3** | GGGCTGGTCGGCCACGTCGGGGTCAGGTTGACT |
| **H5** | GGGGAAGTCAACCTGACCCCGACGTGGCCGAC |
| **H3** | TCAGGCGCCGGGGGCGGTGTCC |
| ***ku*15** | CTGCGTGTCAAGGACTTCAGCAAGCG |
| ***ku*13** | TCAGGCGCCGGGGGCGGTGTCC |
| ***ku*O15** | GCAGCTGCGAAGAAGTCGATGGAAAAGAGAAG |
| ***ku*O13** | CATCTTCTCTTTTCCATCGACTTCTTCGCAGC |
| ***ku*O25** | ACCGCCCCCGGCGCCTGAATCGTCGTAACTCA |
| ***ku*O23** | GTGCCCTGAGTTACGACGATTCAGGCGCCGGG |
| ***ku*23** | AACTGTGTGACGTCGCGGAACTCGTGGC |
| ***sir2*15** | GCGCGTCGTCACCCAGAACATCGACGATCT |
| ***sir2*13** | TCAGGCGCCGGGGGCGGTGTCCG |
| ***sir2*O15** | AACTGCTCAACCGCTCGGCCATGGAAAAGAGAAGAT |
| ***sir2*O13** | CCATCTTCTCTTTTCCATGGCCGAGCGGTTGAGCA |
| ***sir2*O25** | CCCCCGGCGCCTGAACTCTAGGGCCGCA |
| ***sir2*O23** | GGCGGTGCGGCCCTAGAGTTCAGGCGCCG |
| ***sir2*23** | GCACCAACTTCGAGTTCTATCCGTCCACGGT |
