# Supplementary material for: A Sir2-Like Protein Participates in Mycobacterial NHEJ
Source: PLoS One. 2011 May 26;6(5):e20045. doi: 10.1371/journal.pone.0020045 (PMC3102665; doi:10.1371/journal.pone.0020045)
Supplement: Table S2 — Primers used for PCR analysis of TAP-tag knock-in strains. (DOC) [file pone.0020045.s008.doc]

| **Primer** | **Sequence (5′-3′)** |
| --- | --- |
| ***ku*kia** | CGACAAGAGCTACTTCCTTGAGCCCGATTC |
| ***ku*kib** | GTAACAGGGATTCTTGTGTCACAGCGGACC |
| ***ku*kic** | GAGACGGAGGTCATCGAATTGAGTGCTG |
| ***sir2*kia** | CGAAGACCCGCTACCTTGGTTGACGTG |
| ***sir2*kib** | GTAACAGGGATTCTTGTGTCACAGCGGACC |
| ***sir2*kic** | CGTGGCTGTGGAAGACGCTGTCGA |
