# Supplementary material for: A Sir2-Like Protein Participates in Mycobacterial NHEJ
Source: PLoS One. 2011 May 26;6(5):e20045. doi: 10.1371/journal.pone.0020045 (PMC3102665; doi:10.1371/journal.pone.0020045)
Supplement: Table S3 — Primers used for generating the sir2 or ku knock-out cassettes. (DOC) [file pone.0020045.s009.doc]

| **Primer** | **Sequence (5′-3′)** |
| --- | --- |
| ***sir2*15** | CTGCTCGAGTTCGGCTGAGCGCGCCT |
| ***sir2*13** | TCAGGCGCCGGGGGCGGTGTCC |
| ***sir2*O15** | GTTGACGTGCAAGCCCCGACGTGGCCGA |
| ***sir2*O13** | GTCGGCCACGTCGGGGCTTGCACGTCAACCAA |
| ***sir2*O25** | GCCCCCGGCGCCTGAACTCTAGGGCCGCA |
| ***sir2*O23** | GGTGCGGCCCTAGAGTTCAGGCGCCGGGG |
| ***sir2*23** | GCACCAACTTCGAGTTCTATCCGTCCAC |
| ***ku*15** | GACACGAGCGGATCCGTTCCCGGACTT |
| ***ku*13** | TCAGGCGCCGGGGGCGGTGTCC |
| ***ku*O15** | GGAAGGGTTCGATCGCCCCGACGTGGCCG |
| ***ku*O13** | CGGCCACGTCGGGGCGATCGAACCCTTCCAGAT |
| ***ku*O25** | GCCCCCGGCGCCTGAATCGTCGTAACTCAG |
| ***ku*O23** | TGCCCTGAGTTACGACGATTCAGGCGCCG |
| ***ku*23** | AACTGTGTGACGTCGCGGAACTCGTG |
