# Supplementary material for: A Sir2-Like Protein Participates in Mycobacterial NHEJ
Source: PLoS One. 2011 May 26;6(5):e20045. doi: 10.1371/journal.pone.0020045 (PMC3102665; doi:10.1371/journal.pone.0020045)
Supplement: Table S4 — Primers used for PCR analysis of sir2 or ku knock-out strains. (DOC) [file pone.0020045.s010.doc]

| **Primer** | **Sequence (5′-3′)** |
| --- | --- |
| ***sir2*koa** | GCAAGTTCATGCGCACCGTGGTGT |
| ***sir2*kob** | GTAACAGGGATTCTTGTGTCACAGCGGACC |
| ***sir2*koc** | CGTGGCTGTGGAAGACGCTGTCGA |
| ***ku*koa** | ATCCCTCACTGATTCGGCGTCCGTTGT |
| ***ku*kob** | GTAACAGGGATTCTTGTGTCACAGCGGACC |
| ***ku*koc** | GGCAGACAGAACAGCAGGCCAAGC |
