# Supplementary material for: A Sir2-Like Protein Participates in Mycobacterial NHEJ
Source: PLoS One. 2011 May 26;6(5):e20045. doi: 10.1371/journal.pone.0020045 (PMC3102665; doi:10.1371/journal.pone.0020045)
Supplement: Table S5 — Primers used in qRT-PCR. (DOC) [file pone.0020045.s011.doc]

| **Primer** | **Sequence (5′-3′)** |
| --- | --- |
| **rrsA5** | CCGTGAGGTGGAGCGAATC |
| **rrsA3** | TGATCTGCGATTACTAGCGACTC |
| **ku5** | GAGGTCGTCGAGTTCATCC |
| **ku3** | GTCTCAGCGAGCGTCTTG |
| **sir5** | CGTCGTCACCCAGAACATCG |
| **sir3** | ACCCGCAAGCGTCACAAC |
